# Negative and positive masked-priming – implications for motor inhibition

**DOI:** 10.2478/v10053-008-0033-0

**Published:** 2008-07-15

**Authors:** Petroc Sumner

**Affiliations:** Cardiff University, Cardiff, UK

**Keywords:** object updating, active mask, automatic, subliminal

## Abstract

Masked stimuli can prime responses to subsequent target stimuli, causing response benefits when the prime is similar to the target. However, one masked-prime paradigm has produced counter-intuitive *negative* compatibility effects (NCE), such that performance costs occur when prime and target are similar. This NCE has been interpreted as an index of an automatic self-inhibition mechanism that suppresses the partial motor activation caused by the prime. However, several alternative explanations for the NCE have been proposed and supported by new evidence. As a framework for discussion, I divide the original theory into five potentially separable issues and briefly examine each with regard to alternative theories and current evidence. These issues are: 1) whether the NCE is caused by motor inhibition or perceptual interactions; 2) whether inhibition is self-triggered or stimulus-triggered; 3) whether prime visibility plays a causal role; 4) whether there is a threshold for triggering inhibition; 5) whether inhibition is automatic. Lastly, I briefly consider why NCEs have not been reported in other priming paradigms, and what the neural substrate for any automatic motor inhibition might be.

It is well established that backward-masked stimuli can influence subsequent responses to other stimuli, even when the masked stimuli are not consciously perceived (Bar & Biederman, 1998; Enns & Di Lollo, 2000; Forster, Davis, Schoknecht, & Carter, 1987; Leuthold & Kopp, 1998; Marcel, 1983; Neumann & Klotz, 1994). Generally there is a positive compatibility effect, such that responses are facilitated if the ‘prime’ is visually similar to the target or is associated with the same response, or has some cognitive association with the target, relative to the neutral case in which the prime has no association with the target or any response. Conversely, responses are hindered if the primes are associated with a different response. Such priming effects have provided key evidence that non-perceived stimuli can elicit partial activation of motor responses or recognition processes.

While positive priming effects occur in many paradigms, counter-intuitive negative compatibility effects have been recorded under certain circumstances (Aron et al., 2003; Eimer & Schlaghecken, 1998, 2001, 2002; Eimer, Schubö, & Schlaghecken, 2002; Klapp, 2005; Klapp & Hinkley, 2002; Praamstra & Seiss, 2005; Schlaghecken & Eimer, 2000, 2002; Schlaghecken, Munchau, Bloem, Rothwell, & Eimer, 2003; Seiss & Praamstra, 2004; see Eimer & Schlaghecken, 2003, for a review). In this paradigm, participants are generally asked to make speeded button presses to leftward or rightward pointing arrows, which are preceded by masked primes that may be identical to the target arrow (compatible) or identical to the alternative target (incompatible). These primes are often not perceived at all, but they can affect responses to the targets in a biphasic pattern. When the interval between prime and target is very short (0 – 60 ms), performance benefits generally occur on compatible trials and perfor-mance costs on incompatible trials (a positive compatibility effect, PCE). However, when the inter-stimulus interval (ISI) is longer (100 – 200 ms), performance costs can occur on compatible trials and benefits on incompatible trials (a negative compatibility effect, NCE). Such a negative bias has been measured with “free-choice” responses as well as speeded discrimination responses (Klapp & Haas, 2005; Klapp & Hinkley, 2002; Schlaghecken & Eimer, 2004), and it is also reflected in EEG recordings (Eimer & Schlaghecken, 1998, 2003; Praamstra & Seiss, 2005). However, the explanation for the NCE has recently been the subject of extensive debate.

## Threshold-dependent automatic self-inhibition?

The NCE was originally suggested to represent an automatic self-inhibition mechanism that suppresses the partial activation initially caused by the prime (e.g. Eimer & Schlaghecken, 1998; Klapp & Hinkley, 2002; Schlaghecken & Eimer, 2002). In this account, the prime automatically and very quickly causes some activation of the motor mechanisms associated with it, and, through lateral inhibition, some deactivation of alternative responses, leading to a PCE if the target is presented at this stage. However, in the absence of further perceptual evidence supporting such response activation, an inhibitory phase automatically follows so that inappropriate response tendencies do not develop into actual responses. This inhibition of the partially activated response also releases the alternative responses from their partial deactivation, so that an NCE occurs (see Schlaghecken, Bowman, & Eimer, 2006, for a more detailed account). This activation-followed-by-inhibition pattern comfortably explains the transition from PCE to NCE as the target is delayed, and is seen as a characteristic feature of low-level motor-control with wide implications for the production of both voluntary and automatic actions.

The theory has several potentially separable components, and the aim of this paper is to examine them individually rather than to accept or reject the theory as a whole. Most importantly, the NCE is hypothesised to represent motor inhibition, rather than any perceptual interactions that might lead to the reversal of priming without requiring motor inhibition. Second, the inhibition is supposed to occur because the motor system detects unwanted activation – self-inhibition within the motor system – rather than because an external stimulus triggers inhibition. Third, such self-inhibition was suggested to occur due to the lack of continued perceptual evidence for the partially activated response – in other words, whether inhibition occurs is causally related (inversely) to conscious perception of the prime. The inhibitory mechanism is also thought to have a threshold, so that it does not occur for very weak invisible primes that elicit very weak motor activation. Lastly, the whole mechanism is considered to be automatic, rather than driven by any volition to suppress the response.

The original theory has become known as “self-inhibition”, but to avoid potential confusion, it is important to note that some of its components are not captured by this phrase. For example, “self-inhibition” has become synonymous with the view that prime visibility plays a causal role, and has been contrasted with theories that do not give prime visibility any central importance (see below). However, Praamstra and Seiss (2005) proposed an alternative to “self-inhibition” that differed in its dependence on prime visibility, but conformed to my definition of “self”, because it envisaged inhibition that was initiated within the motor system. Thus the debate about prime visibility is logically separable from whether the NCE is caused by self-motor-inhibition or not, and in this review I attempt to treat each issue independently. 

### Motor inhibition or perceptual interactions?

#### Mask-induced priming

An alternative to the main tenet of motor inhibition is that the NCE may instead be produced by positive priming of the alternative response (Lleras & Enns, 2004; Verleger, Jaśkowski, Aydemir, van der Lubbe, & Groen, 2004). These authors remind us that masks do not simply interrupt processing of the prime, but are stimuli in their own right and have the potential to produce their own priming effects. Furthermore, if the mask alters our perceptual representation of the prime, then the prime may alter our perceptual representation of the mask – it is perhaps best to consider the prime-mask sequence as one dynamic stimulus rather than two static ones – and any resultant saliency imbalance for features similar to the targets could potentially cause patterns of response activation opposite to, or different from, those predicted from the primes alone. For simplicity, I refer to this general possibility as mask-induced priming, encompassing Lleras and Enns’ (2004) “object updating” and Verleger et al.’s (2004) “active mask” accounts of the NCE.

In the early studies (e.g. Eimer, 1999; Eimer & Schlaghecken, 1998; Schlaghecken & Eimer, 2000), and in some conditions in subsequent experiments (Klapp & Hinkley, 2002, Experiment 2; Lleras & Enns, 2004, 2005; Verleger et al., 2004) masks were constructed by superimposing the two possible prime stimuli, which were most often leftward and rightward double arrows (<< and >>). This meant that the sequence of presenting, for example, a leftward prime followed by a mask was equivalent to presenting leftward arrows followed by rightward arrows superimposed. The appearance of rightward arrows in the mask could comfortably explain the reversal of the compatibility effect without the need for any inhibition, and it is now generally accepted that when masks are constructed this way, mask-induced priming plays an important role in creating the NCE (Jaśkowski & Przekoracka-Krawczyk, 2005; Klapp, 2005; Lleras & Enns, 2004; Schlaghecken & Eimer, 2002, 2006; Verleger et al., 2004).

Many more recent studies have measured NCEs with masks composed of random lines rather than superimposed arrows (Aron et al., 2003; Eimer & Schlaghecken, 2002; Praamstra & Seiss, 2005; Schlaghecken & Eimer, 2002, 2004; Seiss & Praamstra, 2004; Sumner, Tsai, Yu, & Nachev, 2006) and the appearance of such masks is not obviously equivalent to the appearance of the second prime. However, the random line masks still contain diagonal lines and intersections that could possibly cause mask-induced priming for arrow targets. It is common for the visual system to exaggerate novelty or feature differences, and this being the case, the features in the mask that are not also in the prime may be more salient than the features that are also in the prime, potentially causing priming of the target opposite to the prime – an NCE. To test this, a recent study deliberately introduced physical feature imbalance into random line masks to measure the extent of priming this would create for the responses associated with those features (Sumner, in press). Imbalance of physical features ought to be a stronger source of priming than the suggested imbalance of feature salience created by repetition of prime features in normal random line masks. However, the priming effect from these physically imbalanced masks was much smaller than the size of the NCE, suggesting that mask-induced priming is not the major component of the NCE when random line masks are employed. Furthermore, Klapp (2005) and Schlaghecken and Eimer (2006) have found NCEs using masks composed only of vertical and horizontal lines, or chequer-boards of rectangles, and thus sharing no geometric features with the arrow primes and targets. Therefore it is clear that directional mask-induced priming, produced through salience modulation of geometric features in the masks, cannot fully account for the NCE. However, note that the masks still shared temporal onset features and spatial location with the primes and targets, and these features are also potentially important (Lleras & Enns, 2006), as discussed below.

#### Prime-target perceptual interactions

Although perceptual interactions between geometric features in prime and mask cannot fully account for the NCE, there may be other potential perceptual interactions. Lleras and Enns (2005) suggested that interactions between the prime and the target may render targets opposite to the prime more salient and therefore cause an NCE (through processes akin to repetition blindness or negative priming for example). Eimer (1999) originally attempted to rule out these types of explanation for the NCE, but since that study used masks composed of superimposed primes, a major source of the NCE was likely to have been mask-induced priming and we cannot rely on the results to prove that prime-target interactions are not important in other situations. 

Lleras and Enns (2005, 2006), using masks that did not contain any prime or target features, have found small NCEs only when targets and primes were presented at the same location, and a PCE when they were presented at different locations. This may imply that prime-target interactions play an important role, but there are other possible explanations for the discrepancy. If interactions between prime and target can affect target salience, such interactions might also affect *prime* salience, and thus the latter may not have been constant even though the same primes and masks were used in each condition. More importantly, attention to the prime location may have differed across conditions because participants would have attended to the target locations. Attention has been found to modulate the effect of primes in this paradigm (Sumner et al., 2006) and in others (Lachter, Forster, & Ruthruff, 2004; Naccache, Blandin, & Dehaene, 2002). Previously, Schlaghecken and Eimer (2002) had also reported no NCE for primes presented away from the target position at fixation, but they offered a different explanation in terms of a threshold for motor inhibition, which will be discussed further below. 

Other studies have found robust NCEs with primes and targets presented in different locations (Praamstra & Seiss, 2005; Seiss & Praamstra, 2004; Sumner et al., 2006), while employing random line masks which do not appear to generate much mask-induced priming (Sumner, in press). Moreover, in a control experiment, Sumner et al. (2006) found equivalent NCEs for targets presented in the same location as the prime or in a different location (19 ms vs. 20 ms). Taken together, the current evidence suggests that while perceptual prime-target interactions may occur, they do not account for the NCE measured in most studies. 

#### Source confusion and feature discounting

Huber et al. (2001, 2002) explained a pattern of negative priming measured during a word recognition task with a computational theory for ‘responding optimally with unknown sources of evidence’ (ROUSE). Essentially they argued that feature representations activated noisily by a series of stimuli (e.g. a prime and a target) are subject to source confusion, and when participants are required to recognise only one of the stimuli (the target), they must employ a ‘discounting’ mechanism that estimates the feature activity associated with the prime and removes it from the decision about the target. In standard priming, the discounting mechanism underestimates the prime-related activity and thus allows some prime activity to influence the target decision process. But under some circumstances, the discounting mechanism might overestimate the prime-related activity, and thus overcompensate for features in the prime, resulting in a bias against targets that share these features (i.e. a form of NCE).

Huber et al. found that over-discounting occurred only for clearly visible primes of a long duration or if the prime itself was the target for an action or a decision, and so it would not be expected for the invisible task-irrelevant primes normally associated with the NCE. However, the theory might be extended if participants must discount the features associated with the clearly visible mask, and there is source confusion between the features of the mask and the prime, so the features of the prime also get discounted (J. Enns, personal communication). This idea goes beyond that of Huber et al. because their discounting mechanism was a cognitive process and was not envisaged to be sensitive to differences in feature representations that remain outside conscious perception (i.e. when the difference between the two types of prime-mask combination can cause an NCE, but participants cannot use this feature difference to discriminate the primes). However, even with this extension, the theory fails to explain why physical feature imbalance in the masks should produce positive priming, while invisible primes still produced an NCE (Sumner, in press). If the NCE is to be explained by over-discounting of prime features that are source-confused with the mask, why was there not over-discounting also of the mask features themselves?

### Self-triggered or stimulus-triggered inhibition?

Above, it was concluded that perceptual interactions between primes and masks or primes and targets certainly occur in some stimulus arrangements, but they do not seem to account for the NCE measured in all circumstances (e.g. when random line masks are employed, and the primes and targets do not occur in the same location). Therefore it has been accepted by most researchers that some form of motor inhibition is at play. Most accounts agree on the existence of mutual inhibitory connections between response alternatives, but an additional factor is required to reverse the initially positive effect of the prime (in which the associated response is partially activated and the other response is therefore partially inhibited). However, the mechanism by which this occurs may not follow the original hypothesis developed by Schlaghecken and Eimer (e.g. 2002, 2006) and in Klapp and Hinkley (2002) , in which partially activated responses *self-inhibit* when perceptual evidence supporting them is removed. Praamstra and Seiss (2005) proposed that alternating cycles of activation and inhibition are inherent in the competitive interactions between response alternatives – perhaps due to a mechanism that detects and opposes large activation differences – and do not crucially depend on the presence or absence of perceptual evidence for the primes. However, the role of perceptual evidence will be discussed below, and for the present discussion, Praamstra and Seiss’s theory is also one of self-inhibition because the impulse that reverses the PCE into an NCE comes from within the motor system. A recently proposed alternative is that motor inhibition may be stimulus-triggered (Jaśkowski, 2007; Jaśkowski & Przekoracka-Krawczyk, 2005; Lleras & Enns, 2006; Mattler, 2005). 

Growing evidence suggests that to obtain an NCE, it is essential to have a second stimulus (the “mask”) between the prime and the target, and that this second stimulus does more than simply reduce the visibility of the prime (Jaśkowski, 2007; Jaśkowski & Przekoracka-Krawczyk, 2005; Lleras & Enns, 2006). While no NCEs were found when there was no second stimulus onset after the prime, NCEs did occur with “masks” that did not spatially overlap the primes, and thus left them equally visible (Jaśkowski, 2007; Lleras & Enns, 2006). These findings can be explained by the “mask-triggered inhibition” hypothesis (Jaśkowski, 2007; Jaśkowski & Przekoracka-Krawczyk, 2005) and the “onset-triggered suppression” hypothesis (Lleras & Enns, 2006), in which the appearance of a second stimulus after the prime automatically elicits an “emergency break” or “whoops response” that inhibits motor activation. To explain the NCE, such emergency break inhibition is applied most to the response activated by the prime, rather than symmetrically to all response possibilities. This idea has similarities also to the “cancellation-inhibition” theory developed by Arnold Stoper (see Klapp & Hinkley 2002, p. 266), in which the mask causes any in-progress processing of the prime to be cancelled and any prime-related activation is thereby inhibited. More data will be needed to ascertain the relative importance (if any) of self-inhibition and stimulus-triggered inhibition.

### Causal effect of prime visibility?

The original motor inhibition hypothesis proposed that inhibition occurred only when there was no perceptual information supporting the partial response initiation caused by the prime (Eimer & Schlaghecken, 2002). In other words, the NCE is predicted only for “subliminal” primes. However, several studies have found that NCEs can occur when prime discrimination is above chance (e.g. Klapp, 2005; Klapp & Hinkley, 2002; Lleras & Enns, 2005; Mattler, 2005; Sumner et al., 2006), and, conversely, that PCEs can also occur for a range of levels of prime discrimination performance (see e.g. Lleras & Enns, 2006). Thus the NCE does not appear to be associated with a categorical distinction between conscious and unconscious processes.

However, while the transition between invisible and visible primes appears not to be special, a negative relationship between the size of the NCE and prime visibility has occurred in various experiments across several studies, such that the compatibility effect generally seems to be more negative when primes are less discriminable, and more positive when primes are more discriminable (Eimer & Schlaghecken, 2002; Klapp, 2005; Klapp & Hinkley, 2002; Lleras & Enns, 2004, 2005; Schlaghecken & Eimer, 2006; Sumner et al., 2006). So the question becomes: Is there any direct causal effect behind this repeatedly found relationship, or does it occur because prime visibility and the NCE both correlate with other factors manipulated in these experiments? In many cases the relationship occurred in the context of differences between masks, so it is unclear whether it might be attributed to differences in mask-induced priming (Lleras & Enns, 2004, 2005), or some other factor involving the relative strengths of prime and mask. Additionally, given that more than one process can contribute to NCEs (Klapp, 2005), it is likely that these differ in their dependence on the relative strengths of prime and mask. For example, the motor inhibition component may occur only for relatively weak (often invisible) primes but the mask-induced priming component may occur for both weak and strong (invisible and visible) primes.

Klapp and Hinkley (2002) and Klapp (in press) have analysed the correlation across participants between the size of the NCE and prime discrimination performance, which has the advantage that the physical features of all the stimuli are held constant. They found smaller NCEs for participants with better prime discrimination performance. In this context is it interesting that Friederike Schlaghecken (personal communication) and I have independently observed that there are sometimes a minority of participants who perform much better than expected in prime discrimination, and these same subjects normally produce a PCE rather than an NCE. However, it remains difficult to know the causal under-pinning of any such correlation. 

An alternative way to manipulate prime awareness without changing stimulus properties is through perceptual learning. Schlaghecken, Blagrove, and Maylor (in press) measured NCEs with a weak prime and PCEs with a stronger prime, and then trained the participants to discriminate the weak prime to the same degree as the stronger prime. These newly “visible” primes still produced NCEs, demonstrating that prime discriminability itself was not causally related to whether an NCE or PCE occurred. Kenny Yu and I have also collected some unpublished data using perceptual learning and found no consistent relationship between increased prime discrimination and a change from NCE to PCE. Thus it appears that the apparent correlation reported in many studies between prime visibility and the size of the NCE (or the transition from NCE to PCE) may be due to certain physical stimulus properties of prime and mask, rather than being causally dependent on actual conscious perception of the prime (or on measured prime discrimination performance at least).

However, if conscious awareness does not play a causal role, it remains unexplained why relatively weak primes should be more associated with NCEs than stronger primes. The answer may lie in the relative strengths and differing time courses of motor inhibition and motor activation, both of which may occur to some degree under all circumstances. Inhibition may never fully overcome the initial activation phase caused by a strong prime. Indeed, if the inhibition is stimulus-triggered by the mask (Jaśkowski, 2007; Jaśkowski & Przekoracka-Krawczyk, 2005; Lleras & Enns, 2006), rather than triggered by the motor activation itself, we might not expect it to increase in proportion with prime-related activation.

### Threshold-dependent inhibition and the central-peripheral asymmetry

There is a further complication in the relationship between prime strength and the NCE. When masked primes have been presented in the periphery, rather than at fixation, or when primes at fixation have been degraded, a PCE, not an NCE has occurred (Schlaghecken & Eimer, 2000, 2002, 2006). In these cases, the primes were weaker, not stronger, than the standard primes presented at fixation. This reversal of the relationship between prime strength and the NCE was attributed to a threshold mechanism that does not trigger inhibition unless a threshold of motor activation is reached (Schlaghecken & Eimer, 2002).

Lingnau and Vorberg (2005) systematically varied prime eccentricity and size as well as mask-target SOA, and argued that rather than a threshold below which inhibition does not occur, primes with reduced cortical representation (either through size or eccentricity) simply produce inhibition with a reduced amplitude and slower time course. They point out that when measuring at just one SOA, such delayed and reduced inhibition might be missed. However, this leaves unexplained why a relatively strong early PCE still occurred for primes with weak cortical representation. In other words, why is the inhibition larger than the facilitation for some primes (resulting in an NCE at some SOAs) but only equivalent to the facilitation for other primes (eliminating the PCE but never producing an NCE at any SOA). 

Lleras and Enns (2006) ascribe the central-peripheral asymmetry to the general principal of “repeated location advantage” – that priming is more effective when stimuli are presented in the same location. However, on its own, this principle does not explain why the priming should be more negative rather than more positive. Mask-induced priming or stimulus-triggered inhibition can supply the necessary negative direction for the foveal primes, but then leave unexplained the same issue neglected by Lingnau and Vorberg – why weak or peripheral primes can create a PCE but not an NCE. If the ability of the mask to trigger priming or inhibition is diminished by its peripheral location, why is the ability of the prime to produce positive priming not diminished to an equal degree? We might speculate that the motor activation process triggered by the prime is less spatially selective – in terms of proximity of the stimulus to the expected target location – than is the emergency brake response triggered by the mask. However, even this explanation would not account for the PCEs measured with degraded or small primes rather than peripheral primes (Schlaghecken & Eimer, 2002). The next step in more thoroughly testing the threshold-dependent aspect of the motor inhibition account might be to investigate whether the pattern of results reported so far generalises to all methods of weakening primes, and occurs also with masks that do not share geometric features with the primes.

### Automaticity and volition

One of the attractions of the masked-prime task was that it appeared to be a window into wholly automatic and subconscious motor control processes, whereas most paradigms of motor inhibition rely on the participant’s volitional will to inhibit a certain action. Indeed, the concept of automatic motor inhibition originally proposed (e.g. Eimer & Schlaghecken, 2003) may challenge the traditional distinction between “automatic” mechanisms and “control” processes, because it envisages a motor control process that does not represent top-down executive control. Consistent with the low level and automatic nature of the NCE, it appears to be no different in children and adults, while other types of control seem immature in children (Schlaghecken & Siman, 2006).

Although motor inhibition may occur regardless of prime visibility, as discussed above (e.g. Praamstra & Seiss, 2005), the argument for automaticity does rest on prime visibility. If prime discrimination performance is at chance (i.e., if neither the visual stimuli nor the response tendencies they evoke can be consciously discriminated), then any motor inhibition associated with the direction of the prime cannot be the result of conscious volition. As I understand it, this logic must also apply to the “mask-triggered inhibition” hypothesis (Jaśkowski, 2007; Jaśkowski & Przekoracka-Krawczyk, 2005) and the “onset-triggered suppression” hypothesis (Lleras & Enns, 2006). Although the mask stimulus is perceived, allowing some involvement of conscious volition in a general “emergency break” or “whoops response”, the NCE must be explained by directional inhibition specific to the primed response. If the prime discrimination is at chance, this directional aspect of the inhibition cannot be volitional (the same logic would also apply to the feature discounting mechanism discussed in section 1).

A related question concerns the automaticity of the initial activation elicited by the prime. It has been assumed that the activation-inhibition cycle leading to an NCE occurs only when the stimulus-response (S-R) associations are strong, consistent with the idea of “direct parameter specification” (Neumann & Klotz, 1994). Arrow stimuli provide ‘natural’ associations with left and right responses and seem to most easily elicit robust NCEs (Jaśkowski & Ślósarek, 2007). NCEs have also been reported for other stimuli with left-right asymmetry (Jaśkowski & Ślósarek, 2007; Sumner, in press), but for symmetrical stimuli, which are not so easily associated with left-right responses, only small, if any, NCEs have generally been found, and extensive practice is normally required (Jaśkowski & Ślósarek, 2007; Klapp & Hinkley, 2002; Sumner, in press). However, robust NCEs have recently been reported for button press responses to the emotional expression of face stimuli, which demonstrates that well established S-R associations are not always necessary (Bennett, Lleras, Oriet, & Enns, under review, communicated by J. Enns). This finding may indicate that different rules apply to the different factors contributing to NCEs. Motor inhibition may require well established S-R associations, while mask-induced priming may not. Bennett et al. attribute their result to the latter – a perceptual contrast effect between the emotional prime face and the neutral mask face.

However, to say that motor activation and inhibition is elicited automatically by masked-primes is not to say that it occurs willy-nilly whenever the associated stimulus occurs. Arrow primes have an effect only if the participant currently wishes to respond to arrow targets. If instead the participants must respond right and left to the letters R and L, arrow primes have no effect, despite previous training that associated the arrows with right and left responses (Eimer & Schlaghecken, 1998). Likewise, masked-arrows bias free choice only when free choice trials are interspersed with trials requiring responses to arrows (Klapp & Haas, 2005). Thus the primes must be contained within the set of current “action triggers” (see Kiesel, this volume). Furthermore, Sumner et al. (2006) have argued that non-conscious automatic motor inhibition can be directly modulated by attention. This kind of “conditional automaticity” has also been reported for other priming paradigms (e.g. Kunde, Kiesel, & Hoffmann, 2003, Enns, this volume; Naccache et al., 2002). It has also been reported recently that the NCE is modulated by the proportion of compatible trials, despite participants being unaware of this proportion (Klapp, in press). 

## NCEs in other paradigms?

One puzzle created by the NCE, and a challenge for any theory explaining it, is why NCEs have rarely been found in other masking or priming paradigms. This discrepancy was one inspiration for the mask-induced priming hypothesis (Verleger et al., 2004), but if mask-induced priming is not restricted to the specific case of masks made of superimposed primes, we might expect it in any paradigm in which the mask shares features with the primes and targets (and in order to produce effective masking, masks often do share features with the primes). Mattler (2006, 2007) has recently found NCEs in paradigm in which masked primes preceded cues designating which aspect of a subsequent target was relevant for response. Since these primes were similar to the cues, but not to the targets, they possessed a ‘rule’ association, but no direct motor association. The NCE measured may represent mask-induced priming of the opposite rule because the masks contained the relevant shapes of both primes. However, such NCEs depended critically on having a mask at the appropriate temporal position in the stimulus sequence (Mattler, 2007), which may partially explain why NCEs from mask-induced priming have not been reported more often.

In the case of motor inhibition, if it occurs in this masked-prime paradigm (whether self-triggered or mask-triggered), we must ask why other paradigms do not produce it? One possible answer it that they do, but it is normally not apparent because the parameters needed to reveal it are subtle, and any non-robust and unexpected NCEs discovered may not have been reported. However this answer on its own seems unlikely given the abundance of experiments that have been performed with masked primes of many kinds.

A more plausible possibility is that primes can cause priming in a multitude of ways (e.g. Mattler, 2003, 2005), and only direct motor priming is associated with motor inhibition. Primes may partially activate a variety of representations from purely perceptual representations to motor representations to “deeper” representations, for example, of word meaning. In different paradigms, the relative importance of these different kinds of representation will vary. While the masked-prime paradigm studied here emphasises motor processes (cf. the idea of direct parameter specification, Neumann & Klotz, 1994), word priming, for example, may place most emphasis on recognition processes, and thus not elicit any significant motor priming or inhibition (although it might elicit other negative effects such as feature discounting, Huber et al., 2001). Directional motor inhibition may occur only when priming is based on very simple stimulus-response relationships that efficiently evoke motor initiation. This idea is in keeping with findings, discussed above, that the prime has an effect only when the stimulus-response association is well established and participants currently intend to respond to it. However, given that automatic inhibition processes have been reported for *non-motor* processes in other types of paradigm (e.g. inhibition of return), it remains to be clarified why automatic motor inhibition should not have revealed itself more often in priming experiments.

## Neural substrate

Finally, it is worth briefly considering what the neural substrate of automatic motor inhibition might be (whether it is self-triggered or stimulus-triggered). Behaviourally, the NCE has been found to be effector-specific, such that primes associated with foot movements did not transfer to hand movements and vice versa (Eimer et al., 2002). This suggests that the inhibition operates at an effector-specific motor stage, and the basal ganglia have been implicated because reduced or variable NCEs have been found in patients with Parkinson’s disease or Huntingdon’s disease (Aron et al., 2003; Seiss & Praamstra, 2004), and in an fMRI investigation with healthy subjects, the inhibitory effect was associated with deactivation of the caudate and thalamus (Aron et al., 2003). However, a more recent study reported no difference in the inhibitory pattern between Parkinson’s patients and controls (Seiss & Praamstra, 2006). Meanwhile, absent NCEs were found in two patients with rare small lesions of the supplementary motor area (SMA), which also has effector-specific representations (Sumner et al., 2007). The SMA may therefore mediate automatic inhibition upstream of the basal ganglia.

## Conclusions

In this paper, I have attempted to divide the original interpretation of the NCE – the automatic self-inhibition hypothesis – into five potentially separable components, and to briefly examine each in the light of alternative hypotheses and current evidence. One important consensus emerging among researchers is that more than one mechanism can contribute to NCEs, and the relative importance of these factors depends on the stimulus arrangements employed.

In sum, mask-induced priming is important when masks are composed of features of the prime, but otherwise some kind of motor inhibition is generally accepted to play a key role. Whether this inhibition is self-triggered or stimulus-triggered remains debated; more direct evidence currently exists for the latter idea, but this does not rule out a contribution from self-inhibition. Conscious perception of the prime does not appear to be causally related to the transition of NCE to PCE, but the details of what does cause this transition remain to be explained. The PCEs measured for very weak primes, and the associated threshold component of the motor inhibition hypothesis, also remain debated and intriguing. Lastly, the motor activation and inhibition mechanisms involved in masked priming can be considered automatic, rather than due to top-down or executive control, as long as we accept the notion of “conditional automaticity” – that no automatic mechanism is free from modulation by other factors, and, therefore, whether these “automatic” mechanisms get triggered depends on both external and internal context (including attention and current task ‘goals’).

Thus a component of many measured NCEs appears to represent an automatic effect of motor inhibition, but perceptual interactions can also contribute, and their relative contribution depends crucially on the stimuli used. How any inhibition is implemented and the necessary trigger conditions for it to occur remain debated, and therefore so do the specific reasons why NCEs are measured in some circumstances and not others.
